# Detection of Oral Human Papillomavirus (HPV) and its Clinical Importance

**DOI:** 10.30476/DENTJODS.2021.88338.1326

**Published:** 2022-03

**Authors:** Gabriela Nalli, Paula Mastrotta, María Gabriela Garcia, Silvio Tatti, Sergio Verdú

**Affiliations:** 1 Dept. of Oral Medicine, School of Dentistry, University of Buenos Aires, Buenos Aires, Argentina; 2 Laboratorio Manlab, Área de Filiaciones, Buenos Aires, Argentina; 3 Dept. of Lower Genital Tract, Hospital De Clínicas José de San Martin, Buenos Aires, Argentina

**Keywords:** Human Papillomavirus, HPV, Epithelium, Oral Medicine

## Abstract

**Statement of the Problem:**

Human papillomavirus (HPV) has a tropism for the squamous epithelium and cause a wide range of diseases, from benign lesions to invasive tumors that can affect the oral cavity.

**Purpose:**

This study aimed to estimate HPV infection in compatible stomatological lesions.

**Materials and Method:**

A cross-sectional study was carried out from March 2017 to August 2019, which included patients who attended the Oral Medicine Department of the School of Dentistry of the University of Buenos Aires who presented oral manifestations compatible with HPV infection that accepted to be studied by histopathology and determination of viral genotype by polymerase chain reaction (PCR). The study was carried out from the biopsy fixed in formalin and included in paraffin, for histopathological study and the genotypification of HPV by genotype-specific PCR and/or sequencing of the L1 fragment. To confirm the negative cases hybrid capture method was also used. The 95% OR-IC was estimated.

**Results:**

108 patients, 76 women and 32 men were studied, who underwent a clinical stomatological examination and genotyping of HPV (PCR-specific genotype), being positive for 60 patients and negative for 48. Among the positive cases (n= 60) 46.7% (n= 28) corresponded to elevated lesions infected with high-risk HPV genotypes, 43.3% (n= 26) to elevated lesions with low-risk HPV genotypes, regarding flat lesions it was found that 5% (n=3) corresponded with high-risk HPV genotypes and another 5% (n=3) with low-risk genotypes, with OR 1,076 95% CI (0.1993-5.818). The HPV genotypes found were 2, 6, 11, 13, 16, 18, 26, 31, 32, 33, 35, 51, 58, 64 and 72.

**Conclusion:**

Our results estimated an association between white, bright, and elevated oral lesions and the presence of high-risk HPV.

## Introduction

Human papillomavirus (HPV) is an enveloped double-stranded DNA virus that belongs to the *Papillomaviridae* family [ 1 ]. Human papillomaviruses have a tropism for the squamous epithelium [ 2 ]. HPVs cause a wide range of diseases, from benign lesions to invasive tumors [ 3 ]. This virus is an agent responsible for warts, condylomas, and papillomas at various sites in the body, including the oral cavity [ 4 ]. Scientific evidence suggests that some oral HPV infections may persist, and persistent infection is mandatory for the malignant transformation associated with HPV, however, the progression of virus-induced lesions to malignancy requires additional cofactors [ 2 ].

HPVs are very successful infectious agents. These viruses induce chronic infections that have practically no systemic sequellae [ 5 ].

 Microabrasions or mucosal lesions and the consequent epithelial proliferation mediated by microorganisms and inflammatory cytokines create an ideal microenvironment for initial HPV infection and its subsequent persistence, increasing the risk of transmission and its carcinogenic potential [ 6 ]. HPV is spread by direct cell-to-cell contact without the classic signs of viremia [ 7 ]. After entering the cell, the viral genome is taken to the cell nucleus where it is translated and transcribed. The replication of the viral genome follows some stages; at first stage, E1 and E2 early proteins are synthesized. As a result, 10 to 200 copies of the genome are replicated per cell. Then, during the cell cycle, replication occurs in descendant cells at the same rate. The expression of the E6 and E7 genes leads to cellular transformation or differentiation. Cells begin to divide faster, leading to the formation of benign tumors. At this point, the virus proliferates in the tissue without destroying the cell that harbors it. In the third stage, known as the production stage, the E1 and E2 proteins begin to produce large amounts of viral deoxyribonucleic acid (vDNA). On the other hand, late proteins (L1 and L2) are produced, which serve for the assembly of the new virus. Finally, more superficially localized keratinocyte viruses are released [ 8 ].

The development of premalignant lesions and certain cancers is associated with high-risk HPVs due to their high oncogenic potential. Previous studies of HPV have classified types 16, 18, 31, and 33 as carcinogenic to humans (group 1), type 68 as probably carcinogenic (Group 2A), and other types like 23, 53, 66, 67, 70, 73, and 82 as possibly carcinogenic (Group 2B) (9). Oral HPV infections have been related to sexual behavior, but recent evidence supports their horizontal mouth-to-mouth transmission. Most HPV infections in babies are acquired vertically from the mother during the intrauterine period, during childbirth, or later through saliva [ 2 ].

## Materials and Method

This study was part of the "Rodolfo Erausquin" Clinical Research Support Program of the School of Dentistry, University of Buenos Aires, Argentina. The study included healthy patients with oral lesions compatible with HPV infection that were analyzed using the nested polymerase chain reaction (PCR) and subsequent sequencing. This project was approved by the Institutional Ethics Committee of the Faculty of Dentistry of the University of Buenos Aires in 2017 Resolution (CD) No. 628. A descriptive cross-sectional study was carried out including 108 adult patients from genders, 76 women and 32 men who attended the Department of Oral Medicine of the School of Dentistry of the University of Buenos Aires in the period between March 2017 and August 2019 who had lesions in the oral cavity that were clinically compatible with HPV infection. Patients of legal age who signed the informed consent were included, and immunosuppressed patients or patients who were taking retroviral or immunosuppressive medications were excluded.

### Data collection

All the data was collected in a protocolled medical history that included age, sex, clinical characteristics of the lesion, and its location. Laboratory tests (blood count, coagulogram, liver function test, glycemia, uremia, uricemia, erythrocyte sedimentation) were requested from all patients to assess their general condition. 

### Assessment of oral lesions

The evaluation of the stomatological clinical lesions was carried out by stomatologists and the OPMI PICO S100 CARL ZEISS clinical operating microscope was used. The lesions were observed at 1: 6 in a magnification sequence. The clinical lesions compatible with HPV infection were classified into two groups: flat and elevated. 

**Figure 1:**
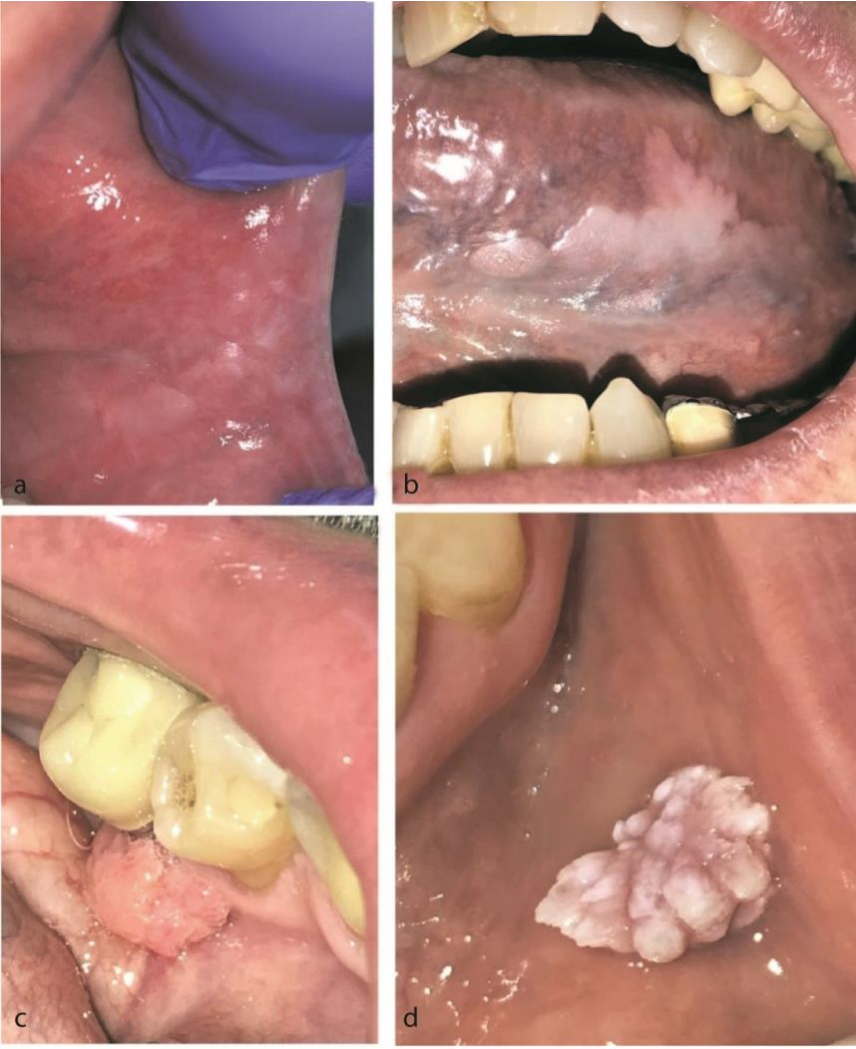
**a:** White spot located in the buccal mucosa. **b:** Keratosis on the left border and ventral face of the tongue. **c:** Vegetation in lingual gingiva of 3.5. **d:** Wart on the buccal mucosa of the lower lip

Among the flat elementary lesions, we found the white spot (Figure 1a); among the elevated lesions, we found keratosis (Figure 1b), vegetations (Figure 1c) and warts (Figure 1d). Regarding the appearance of the lesions, we observed that they could be shiny or translucent (these are white lesions with leukoedema, when dried they do not modify their aspect, they remain bright) and opaque.

### Sample Collection

Biopsies were performed in the affected areas and divided into two parts, one was fixed in 10% formalin, included in paraffin, and stained with hematoxylineosin for its pathological study and the other was stored in saline solution for its determination of HPV, which was analyzed by nested PCR and subsequent sequencing using MY09 /11 and GP05+/06+ primers. It was incubated with 200ul of lysis buffer (Lysis tissue buffer, Roche) and 20ul of proteinase K with shaking at 650 RPM in a thermostatic bath for 12 hours [ 10 ]. 

Subsequently, DNA extraction from this lysate was performed automatically with the MagnaPure 96 kit (Roche) according to the manufacturer's instructions.

To estimate the integrity of the DNA obtained and the absence of PCR inhibitors, a gene fragment of the constitutive gene of β-Globin was amplified in all the samples studied, with the universal oligonucleotides BG1 / BG2 1 by the real-time PCR technique on the LightCycler ® 2.0 Real-Time PCR System (Roche).

The samples where β-Globin amplification was obtained were subjected to viral DNA detection by PCR (primary PCR), using the universal oligonucleotides MY09 / MY11, which amplify a 450 bp fragment in the conserved region of the HPV L1 gene [ 11 - 12 ]. To increase the sensitivity, all the samples that were negative to the amplification with MY09 / MY11 were subjected to a second amplification (secondary PCR) with the universal oligonucleotides (GP5/GP6), located within the sequence recognized by the oligonucleotides. MY09 / MY11 and that amplify a 150 bp fragment [ 12 ]. Both PCR reactions were performed on a Veriti® Thermal Cycler (Thermo Fisher Scientific). A detectable sample for HPV type 16 was used as a positive control and a negative sample for HPV as a negative control. Amplification products were visualized on a 2% agarose gel with a transilluminator.

Capillary electrophoresis of the amplification products resulting from the first and second PCR reactions was carried out, following a sequence reaction with the GP5+ / GP6+ primers, in the ABI® 3500 Genetic Analyzer (Applied Biosystems) sequencer.

Sequence alignments were performed against the reference HPV L1 gene sequences stored in the GenBank database, by BLAST analysis for final validation of the HPV genotype.

To confirm the negative cases, an adaptation of the hybrid capture methodology (HR-HPV DNA, Qiagen, Hilden, Germany) was carried out to detect HPV in biopsies.

DNA extraction was performed as described for subsequent amplification by PCR and the extract obtained was placed in the Digene HC2 DNA collection device. The rest of the procedure was carried out according to the manufacturer's instructions. “HC2 is a capture molecular hybridization assay that utilizes chemiluminescent detection to provide a semi-quantitative result. The assay is calibrated to detect approximately 4,700 genome equivalents (or 1pg/ml) of target HPV, represented by an RLU (relative light unit) measurement greater than or equal to the cutoff value calculated in each run by a series of standards. A measurement less than the cutoff was scored as negative. The samples were analyzed for the presence of HR HPV types 16, 18, 31, 33, 35, 39, 45, 51, 52, 56, 58, 59 and 68. Positive and negative controls (provided by the manufacturer) were included in each run (Sandri M. *et al*., 2006) [ 13 ].

## Results

**Figure 2:**
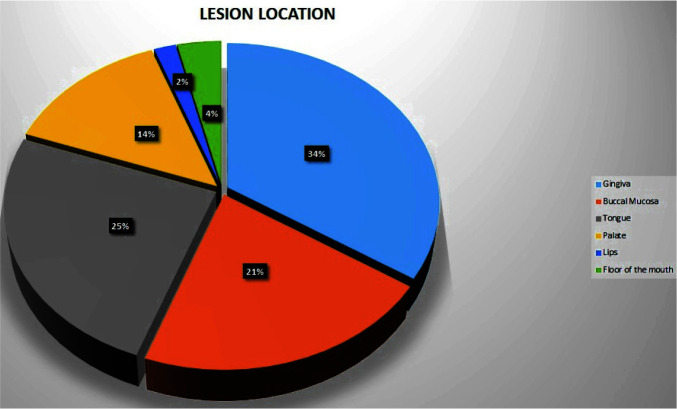
Location of oral lesions

108 patients, 76 women, and 32 men, aged between 23 and 83 years old, with a mean age of 54, a median of 56, and a mode of 63, were studied, who underwent a clinical and histopathological examination and HPV genotyping of their oral lesions (PCR-specific genotype), being positive for 60 patients and negative for 48. Within the sample (n=108), 78% (n=84) presented elevated lesions (keratosis, vegetations and warts) and 22% (n=24) flat lesions (white spot). Regarding their appearance, it was observed that 61% (n= 66) corresponded to bright or translucent lesions and 39% (n=42) to white opaque, or pink lesions. The most frequent location was gingiva in 34% of cases (n=37) and tongue 25% (n= 27) (Figure 2). The prevalence of oral HPV in our sample was 56% and we found high and low-risk HPV genotypes.

**Figure 3:**
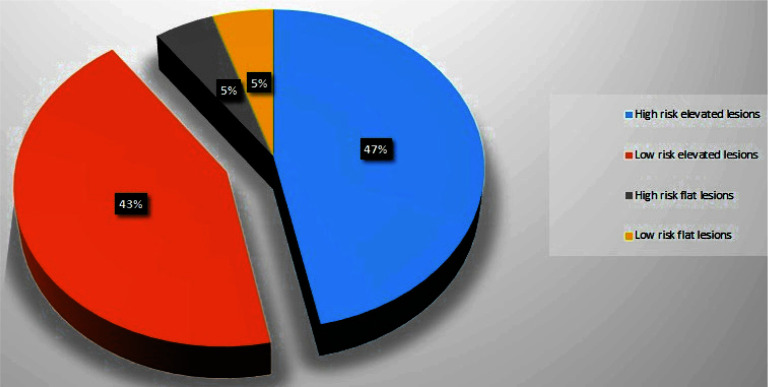
% of low and high-risk human papillomavirus genotypes present in flat and elevated lesions

Among the positive cases (n= 60), 47% (n= 28) corresponded to elevated lesions infected with high-risk HPV genotypes, 43.3% (n= 26) to elevated lesions with HPV genotypes of low risk, in terms of flat lesions it was found that 5% (n=3) corresponded to high-risk HPV genotypes and another 5% (n=3) with low-risk genotypes. Being the OR 1,076 95% CI (0.1993-5,818) (Figure 3). 

The HPV genotypes found were 2, 6, 11, 13, 16, 18, 26, 31, 32, 33, 35, 51, 58, 64 and 72. The most frequent high- risk genotype was HPV16 13% (n= 8) and the most frequently presented low-risk genotype was HPV11 20% (n= 12). Within the sample, four patients had coinfection with two types of HPV in the same lesion 6/35, 16/18, 6/11, 11/16.

**Figure 4:**
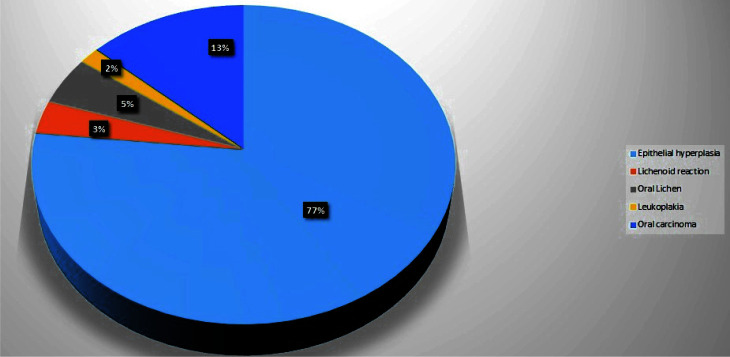
Histopathological diagnoses related to human papillomavirus infected lesions

Histopathological diagnoses associated with HPV- infected lesions corresponded to epithelial hyperplasia 77% (n=46), oral carcinoma 13% (n=8), oral lichen 5% (n=3), lichenoid reaction 3% (n=2) and leukoplakia 2% (n=1) (Figure 4).

All patients with a diagnosis of high-risk HPV continue to be followed up to this day.

## Discussion

A stomatologist makes the first clinical presumption of the presence of HPV in the oral mucosa through the identification of certain elemental lesions that were described in this study and that has long been related to this type of infection. However, confirmation of this clinical diagnosis by pathological study and detection of the type of HPV present in the lesion is necessary [ 11 , 14 ]. Regarding the different types of lesions observed in the stomatological examination authors such as C. Allen and Fornatora describe a subtype of oral epithelial dysplasia with unique clinical and histological characteristics that would probably have a predictive value for the presence of HPV. These are white, flat, slightly elevated, or papillary lesions that have histologic features similar to common dysplasia, such as proliferation and maturation of basal cells, nuclear pleomorphism, increased number of mitoses, nuclear prominence, increased ratio of the cytoplasmic nucleus, and atypical mitotic figures [ 15 ]. Koss and Durfee[ 16 ] introduced the term "koilocytic atypia" to describe cervical lesions that were characterized microscopically by large epithelial cells, with a relatively small and irregular nucleus that was surrounded by a clear glycogen negative halo.

Koilocytes are now known to be pathognomonic for HPV infection and that these cellular abnormalities represent the harmful cellular effect of viral reproduction [ 16 ]. In this article, we made a detailed description of elementary stomatological lesions with clinical inspection and augmentation, where we correlated high and low-risk types with observable clinical lesions. It is of the utmost importance to study the characteristics of the lesions caused by high-risk HPV since this allows us to identify which ones may turn into cancer. Furrer *et al*. [ 17 ] reported that the clinical features considered signs of suspected HPV infection in the oral mucosa were bright white flat lesions with slightly elevated plaques and frankly warty lesions. 

No other authors found in the literature took these oral lesions as a reference as suspects of HPV infection. Like Ribeiro M *et al*.[ 18 ], to improve the detection of HPV, we used the nested PCR technique that uses more than one pair of primers. Therefore, we were able to detect the virus even at very low concentrations. High-quality DNA is required for this technique to reach its optimal conditions [ 18 ]. PCR amplification of HPV DNA is a target amplification technique that is capable of amplifying traces of DNA sequences in a biological sample containing heterogeneous cell types. This technique is very sensitive but also it has low specificity and clinical samples are very prone to cross-contamination. To minimize this adverse effect, their processing must follow meticulous precautions [ 19 - 21 ]. 

In this study, we found the presence of oral infection by multi-type HPV (2 types) in 7% of the patients (n= 4) and most of them presented high-risk HPV genotypes. Bui *et al*. [ 22 ] reported that the prevalence of multiple oral HPV infection (2 to 6 types) was 1.5% (2.5% for men, 0.4% for women). The majority of oral multitype HPV cases (83.8%) harbored one or more oncogenic types [ 22 ]. In this study, the prevalence of HPV was 56% and 13% corresponded to HPV 16. Gillison *et al*.[ 23 ] state that the prevalence of oral HPV infection among men and women between the ages of 14 and 69 in the United States was 6.9% and HPV type 16 was 1% [ 23 ]. Sonawane *et al*. [ 24 ] postulated that the general prevalence of oral HPV infection was 11.5%. Many studies are addressing the presence of HPV in the oral cavity of healthy, uninjured patients. Our study considered the clinical stomatological lesions compatible with HPV infection. This is why the prevalence of this study was higher than that of the studies cited above.

## Conclusion

PCR is a very important technique to determine the HPV genotype present in these lesions due to its high sensitivity. We found an association between high-risk HPV and elevated white lesions, these oncogenic HPVs being a risk factor for the development of these lesions. Further studies need to be made to assess the clinical and molecular implications of the virus in other lesions and the carcinogenic process.

## References

[1] Bzhalava D, Eklund C, Dillner J ( 2015). International standardization and classification of human papillomavirus types. Virology.

[2] Syrjänen S ( 2018). Oral manifestations of human papillomavirus infections. Eur J Oral Sci.

[3] Munoz FX, Bosch S, Desanjose R, Herrero X, Castellsague KV, Shah PJ, et al ( 2003). Epidemiologic classification of human papillomavirus types associated with cervical cancer. N Engl J Med.

[4] Syrjanen K, Gissman L, Koss K (1987). Papillomaviruses and human disease.

[5] Stanley MA ( 2012). Epithelial cell responses to infection with human papillomavirus. Clin Microbiol Rev.

[6] Liu X, Ma X, Lei Z, Feng H, Wang S, Cen X, et al ( 2015). Chronic inflammation-related HPV: a driving force speeds oropharyngeal carcinogenesis. PLoS One.

[7] Castro TM, Bussoloti Filho I, Nascimento VX, Xavier SD ( 2009). HPV detection in the oral and genital mucosa of women with positive histopathological exam for genital HPV, by means of the PCR. Braz J Otorhinolaryngol.

[8] Esquenazi D, Bussoloti Filho I, Carvalho Mda G, Barros FS ( 2010). The frequency of human papillomavirus findings in normal oral mucosa of healthy people by PCR. Braz J Otorhinolaryngol.

[9] IARC Working Group on the Evaluation of Carcinogenic Risks to Humans ( 2007). Human papillomaviruses. IARC Monogr Eval Carcinog Risks Hum.

[10] Ratech H, Masih A ( 1993). Sensitive detection of clonal antigen receptor gene rearrangements in non-hodgkin’s malignant lymphoma with an anchored polymerase chain reaction-based strategy. Am J Clin Patho.

[11] Schneider A, Meinhardt G, De-Villiers EM, Gissmann L ( 1987). Sensitivity of the cytologic diagnosis of cervical condyloma in comparison with HPV-DNA hybridization studies. Diagn Cytopathol.

[12] Resnick R, Cornelissen M, Wright D, Eichnigen GH, Fox HS, Schegget J, et al ( 1990). detection and typing of human papillomavirus in archival cervical cancer specimens by DNA amplification with consensus primers. J Natl Cancer Inst.

[13] Sandri MT, Lentati P, Benini E, Dell'Orto P, Zorzino L, Carozzi FM, et al ( 2006). Comparison of the Digene HC2 assay and the Roche AMPLICOR human papillomavirus (HPV) test for detection of high-risk HPV genotypes in cervical samples. J Clin Microbiol.

[14] Syrjanen SM (1987). Human Papillomavirus infection in the oral cavity. In: Syrjanen KJ, Gissmann L, Koss LG (eds) Papillomaviruses and Human Disease.

[15] Fornatora M, Jones AC, Kerpel S, Freedman P ( 1996). Human papillomavirus-associated oral epithelial dysplasia (koilocytic dysplasia): an entity of unknown biologic potential. Oral Surg Oral Med Oral Pathol Oral Radiol Endod.

[16] Koss LG, Durfee GR ( 1956). Unusual patterns of squamous epithelium of the uterine cervix: cytologic and pathologic study of koilocytotic atypia. Ann N Y Acad Sci.

[17] Furrer VE, Benitez MB, Furnes M, Lanfranchi HE, Modesti NM (2006). Biopsy vs. superficial scraping: detection of human papillomavirus 6, 11, 16, and 18 in potentially malignant and malignant oral lesions. J Oral Pathol Med.

[18] Ribeiro MG, Marcolino LD, Ramos BR, Miranda EA, Trento CL, Jain S, et al ( 2017). High prevalence of human papillomavirus (HPV) in oral mucosal lesions of patients at the Ambulatory of Oral Diagnosis of the Federal University of Sergipe, Northeastern Brazil. J Appl Oral Sci.

[19] Mirghani H, Amen F, Moreau F, Guigay J, Ferchiou M, Melkane AE, et al ( 2014). Human papilloma virus testing in oropharyngeal squamous cell carcinoma: what the clinician should know. Oral Oncol.

[20] Weinberger PM, Yu Z, Haffty BG, Kowalski D, Harigopal M, Brandsma J, et al ( 2006). Molecular classification identifies a subset of human papillomavirus-associated oropharyngeal cancers with favorable prognosis. J Clin Oncol.

[21] Smeets SJ, Hesselink AT, Speel EJ, Haesevoets A, Snijders PJ, Pawlita M, et al ( 2007). A novel algorithm for reliable detection of human papillomavirus in paraffin embedded head and neck cancer specimen. Int J Cancer.

[22] Bui TC, Tran LT, Thai TN, Shete SS, Vidrine DJ, Sturgis EM ( 2017). Prevalence of and risk factors for oral human papillomavirus infection with multiple genotypes in the United States. Sex Transm Dis.

[23] Gillison ML, Broutian T, Pickard RK, Tong ZY, Xiao W, Kahle L, et al ( 2012). Prevalence of oral HPV infection in the United States, 2009-2010. JAMA.

[24] Sonawane K, Suk R, Chiao EY, Chhatwal J, Qiu P, Wilkin T, et al ( 2014). Oral human papillomavirus infection: differences in prevalence between sexes and concordance with genital human papillomavirus infection. Ann Intern Med.

